# An efficient mutagenesis system to improve the propamocarb tolerance in *Lecanicillium lecanii* (Zimmermann) Zare & Gams

**DOI:** 10.3389/fmicb.2023.1243017

**Published:** 2023-09-06

**Authors:** Yanjun Zhang, Xiao Zhang, Weiliang Qiu

**Affiliations:** ^1^Agro-Environmental Protection Institute, Ministry of Agriculture and Rural Affairs, Tianjin, China; ^2^National Pesticide Engineering Research Center, Nankai University, Tianjin, China; ^3^Institute of Plant Protection, Chinese Academy of Agricultural Sciences, Beijing, China

**Keywords:** entomopathogenic fungi, mutagenesis, fungicide tolerance, biological control, pest insects

## Abstract

*Lecanicillium lecanii* (Zimmermann) Zare & Gams is used as an effective biopesticide for the control of sap-sucking insect pests on agricultural crops. However, low fungicide tolerance limits its large-scale field application. To improve the propamocarb tolerance in *L. lecanii*, a composite mutagenesis system was established by using UV-light (U), N-Methyl-N′-nitro-N-nitrosoguanidine (NTG) (N) and N^+^ ion-beam (I). The permutation type of three agents was a consecutive mutagenesis treatment (I/N/U) after an intermittent treatment (U + N + I). The “U” mutagenesis was performed at 254 nm for 60 s and at a distance of 45 cm under a 20 W germicidal lamp, the “N” mutagenesis was performed at a concentration of 1.0 mg/mL NTG for 60 min, and the “I” mutagenesis was performed by low energy N^+^ ion-beam using a dose of 10 × 10^13^ ions/cm^2^ at 30 keV. This composite mutagenesis system was recorded as the “U + N + I + I/N/U,” and then the mutagenesis efficiency in improving propamocarb tolerance was assessed by analyzing changes of mutants in the propamocarb sensitivity, mitotic stability, mycelial growth speed on plates or in liquid, sporulation on plates or aphids, conidial germination, 50% lethal concentration (LC_50_) and 50% lethal time (LT_50_) to aphids, lipid constituent and cell membrane permeability and control against aphids in the presence or absence of propamocarb. Compared to the wild-type isolate with a 50% effective concentration (EC_50_) value of 503.6 μg/mL propamocarb, the Ll-IC-UNI produced by the “U + N + I + I/N/U” had the highest EC_50_ value of 3576.4 μg/mL and a tolerance ratio of 7.1. The mutant was mitotically stable in 20-passage cultivation and did not show any unfavorable changes in growth and virulence indicators. The mutant showed the highest ability to resist or avoid the damaging effects of propamocarb as reflected by the alternations of lipid constituents and membrane permeability. The interval time for applying fungal agent was significantly shortened in this mutant after spraying a field recommended dose of 550 μg/mL propamocarb. In conclude, the “U + N + I + I/N/U” composite mutagenesis mode was efficient and useful to improve the propamocarb-tolerance of *L. lecanii* and the obtained Ll-IC-UNI could have commercial potential for field application.

## Introduction

Entomopathogenic fungi are considered as efficient biological control agents with broad host range and promising to be developed into commercial biopesticides against pest insects. Among them, *Lecanicillium lecanii* (Zimmermann) Zare & Gams, *Beauveria bassiana* (Balsamo-Crivelli) Vuillemin, *Metarhizium anisopliae* (Metschnikoff) Sorokin and *Isaria fumosorosea* Wize were the most commonly studied fungal species (Bamisile et al., 2021). The most important aphid pests for high-value ornamental and vegetable crops were the “melon or cotton aphid” *Aphis gossypii* (Glover), “green peach aphid” *Myzus persicae* (Sulzer) and “potato aphid” *Macrosiphum euphorbiae* (Thomas) (Blümel, 2004). *L. lecanii* had been reported causing natural epizootics in aphid population in tropical and sub-tropical regions of the world (Shah and Pell, 2003), and also were efficient biocontrol agents of the “tea thrips” *Scirtothrips bispinosus* (Bagnall) (Sankara Rama Subramaniam et al., 2021). In laboratory conditions, *L. lecanii* isolates were more virulent compared to other genera belonging to the order Hypocreales (Ascomycota), as well as *B. bassiana*, *Paecylomyces* spp. and *M. anisopliae* against aphid species (Loureiro and Moino, 2006; Asman, 2007). Differences in virulence of *L. lecanii* to different aphid species were found among different isolates (Van Hanh et al., 2007; Diaz et al., 2009). A study had revealed the possibility of applying *L. lecanii* in combination with an aphid alarm pheromone and sublethal doses of the insecticide imidacloprid as part of an autodissemination strategy to enhance the efficacy of the fungus for aphid biocontrol (Hartfield et al., 2001). However, it is not effective in the presence of high fungicide residues, and may be ineffective in the presence of synthetic fungicides. It was reported that the triazols fungicides difenoconazol and tebuconazol were very toxic to *L. lecanii* because 100% inhibition of the mycelial growth, spore production and germination were determined at the concentration of 10 mg/kg fungicides (Castellanos et al., 2012). Therefore, the low tolerance of *L. lecanii* to chemical fungicides impedes its use in integrated pest management (Behle and Birthisel, 2014).

Conidial germination and host infection for entomopathogenic fungi require relatively high humidity, which also facilitates plant diseases in greenhouses. To control the most destructive diseases by Oomycota on vegetable crops, formulations composed of a carbamate fungicide, propamocarb, were used worldwide (Schmuck and Mihail, 2004; Wu et al., 2013), e.g., by leaf-spraying to control downy mildew by *Plasmopara* spp. and root-drenching to control mold by *Phytophthora* spp. Propamocarb interferes with membrane constituent biosynthesis, resulted in unusual fatty acids accumulation, and inhibited mycelial growth, sporangia formation and germination (Thomas et al., 2018). A previous study showed that the wild-type isolate of *L. lecanii* had an EC_50_ value of 503.6 μg/mL propamocarb (Xie et al., 2018a). When used to control pest insects, *L. lecanii* may be exposed to propamocarb residues due to the application of propamocarb once a week at the field recommended dose of 550 μg/mL.

To enhance the fungicide-tolerance of a fungus, several strategies had been developed, including protoplast fusion (Song et al., 2011a), chemical induction (Xie et al., 2017), ultraviolet irradiation (Hassan, 2011), ion-beam irradiation (Shinohara et al., 2013) and genetic engineering (Zhang et al., 2014). The genetic engineering method can result in specific mutagenesis, but requires precise knowledge of the structure and regulation of the fungicide-resistance genes. Unfortunately, our knowledge of the mechanism of propamocarb resistance in fungi is incomplete. Single random mutagenesis methods, like ion-beam irradiation, have been effective for enhancing benomyl-resistance of an *Isaria fumosorosea* isolate (Shinohara et al., 2013). However, this high effectivity only functions to enhance fungicide resistance conferred by single-point gene mutation. Ion-beam irradiation is less effective as another random mutagenesis method (chemical induction or ultraviolet irradiation) when applied to enhance fungicide resistance caused by multiple-point mutation of a function gene, mutation of several function genes and mutation of regulation genes. For instance, propamocarb resistance of entomopathogenic fungus *L. lecanii* was increased by single use of ultraviolet irradiation and propamocarb resistance of nematophagous fungus *L. attenuatum* was increased by single use of chemical induction or ion-beam irradiation in our previous studies (Xie et al., 2017, 2018a,b). Although random mutagenesis has some limitations, there is a significant benefit in that the use of mutants created by random mutagenesis is not subjected to the restrictions applied to genetically modified mutants (Trovão et al., 2022). Moreover, the combined use of two random mutagenesis methods, like chemical induction and ultraviolet irradiation, have been successfully applied to generate mizoribine producing mutants of *Eupenicillium* spp. (Zhang et al., 2010) and cellulose-degrading enzymes producing mutants of *Aspergillus terreus* (Kumar et al., 2015). These results indicate that the combined use of several mutagenesis methods may be a more efficient strategy for improving the fungicide-tolerance of a biocontrol fungus because each technique has a unique mode of action in mutagenesis.

It remains unclear how the microbes respond to each agent used in composite mutagenesis and what the interaction would be between these agents, although several protocols have been recommended for single mutagenesis of a given fungus. In addition, it may be difficult to assess the mutagenesis efficiency based on a few biological characteristics due to pleiotropy of the mutagenesis. In this study, multiple-agent composite mutagenesis systems were constructed for the entomopathogenic fungus *L. lecanii* based on chemical induction, ultraviolet irradiation and ion-beam irradiation, and the whole process and parameters were optimized. After fully considering changes in biological, biochemical and metabolic characteristics associated with propamocarb tolerance of the fungus, we evaluated the efficiency of various mutagenesis systems for improving propamocarb tolerance of *L. lecanii*. Finally, performance of the improved isolates for the control of aphids was determined in the presence of propamocarb. The aims of this study were to: (1) develop an efficient random mutagenesis mode to improve the propamocarb tolerance in *L. lecanii*; (2) create propamocarb-tolerant mutants having commercial potential for field application.

## Materials and methods

### Fungal isolate, chemicals and aphids

A wild-type fungal isolate No.8453 was collected from infected aphids at a tea garden (26°1′23″ N, 119°7′36″ E) in Fuzhou, and single-spore isolated in our laboratory. It was identified as *Lecanicillium lecanii* (Zimmermann) Zare & Gams by the combination of morphological and molecular data (Diaz et al., 2009), and deposited in the China General Microbiological Culture Collection Center (CGMCC). Conidial suspension was prepared by adding 0.05% sterile solution of Tween-80 (Aladdin, Shanghai Aladdin Biochemical Technology Co., Ltd., Shanghai, China) and rubbing surface of the actively growing culture on PDA medium (200 g potato, 20 g glucose, and 18 g agar per liter of distilled water) as described previously (Xie et al., 2018a). The suspension was filtered through 200 mesh sterile nylon net to eliminate mycelia.

Technical grade (98.0%) propamocarb-hydrochloride (Tianlong Biotechnology Co., Ltd., Hangzhou, China) was dissolved in sterile water. Technical grade (95.0%) N-Methyl-N′-nitro-N-nitrosoguanidine (NTG) (J&K Scientific Co., Ltd., Beijing, China) was dissolved in acetone, and then diluted with sterile phosphate buffer (50 mM, pH 6.0). Stock solution of 100 mg/mL propamocarb-hydrochloride and 20 mg/mL NTG were stored at 4°C in darkness.

*A. gossypii* were collected from fields (39°30′36″ N, 116°36′28″ E) that had not been treated with foliar insecticides in April 2018 in Langfang. A colony of aphids, unparasitized and uninfected with entomopathogenic fungi, was initiated on potted plants of cucumber (*Cucumis sativus* L. cv. Shenchun) in cages held at 24 ± 1°C and a photoperiod of 16 L:8D until a sufficient number of aphids were available to perform a bioassay in August 2018. To synchronize aphid age for use in the bioassay was conducted as previously (Xie et al., 2018a). A group of adult aphids was placed on an 8 cm cucumber leaf disc in a petri dish containing wet filter paper and held for 18 h at 24 ± 1°C and a photoperiod of 16 L:8D. Adult aphids were removed from the leaf disc, and the progeny was then transferred to potted cucumber plants and incubated for 6 days in an insect rearing room (24 ± 1°C, 16 L:8D) before the bioassay.

### Single-agent mutagenesis and mutant selection

Cells exposed to NTG, UV light or N^+^ ion-beam can occur DNA damage both directly and through the generation of reactive oxygen species, leading to single nucleotide changes and/or deletion (Song and Yu, 2000; Ikehata and Ono, 2011; Li et al., 2013; Bose, 2014). Based on the mechanism of mutagenesis, NTG (N), UV-light (U) and N^+^ ion-beam (I) were chosen, and the optimized parameters for *Lecanicillium* spp. in previous studies were adopted.

UV mutagenesis was completed by UV-C irradiation at 254 nm for 60 s with a 20 Watt germicidal lamp (Beijing Haoteguang Ultraviolet Radiation Technology Co., Ltd., Beijing, China) placed at the distance of 45 cm from sterile glass petri dishes (90 mm diameter) (Bomex, BOMEX (Yongqing) Glass Co., Ltd., Langfang, China) as described previously (Xie et al., 2018a). A 100 μL aliquots of conidial suspension (10^3^ conidia/ml) was equally spread on PDA medium. The conidia on plates were exposed to UV-C light at 254 nm for 60 s. Irradiation-treated and-free plates were immediately packed with black cloth, and incubated at 25°C for 5 d. Number of single colonies was measured in five replicated plates. Conidial lethality rate was calculated by the formula:conidial lethality rate = (1 - average number of colonies on treated plates/average number of colonies on control plates) × 100%.

Chemical mutagenesis was completed by NTG induction at the concentration of 1.0 mg/mL for 60 min as described previously (Xie et al., 2017). Stock solution of NTG was mixed with conidial suspension (10^8^ conidia/ml), then the mixture was diluted to final NTG concentration of 1 mg/mL with sterile phosphate buffer (50 mM, pH 6.0) and the culture was incubated at 25°C for 60 min. Mutagenesis was stopped by diluting 100-fold immediately with sterile phosphate buffer (50 mM, pH 6.0). The suspended sample was centrifuged at 5000 rpm for 10 min. Then the conidia were harvested and washed three times with sterile distilled water to remove traces of NTG. The conidial pellet was diluted in sterile phosphate buffer (50 mM, pH 6.0) and final conidial concentration was adjusted to 10^3^ conidia/ml. Conidial suspension was spread on PDA medium and the plates were incubated at 25°C for 5 d. Conidial lethality rate was calculated as described for the UV mutagenesis.

Ion-beam mutagenesis was completed by low energy N^+^ ion-beam irradiation using the dose of 10 × 10^13^ ions/cm^2^ at 30 keV with a LC-4 ion implanter at the Institute of Semiconductors, Chinese Academy of Sciences as described previously (Xie et al., 2018b). 0.5 mL conidial suspension (10^8^ conidia/ml) was spread on a sterile glass petri dish (60 mm diameter), and then air-dried on a sterile operating table with sterile air until membrane formed. The dish was placed on the sample holder, and then implanted with the energy of 30 keV, irradiation dose of 10 × 10^13^ ions/cm^2^, pulse frequency of 6.25 × 10^11^ ions/cm^2^s and chamber vacuum of 10^−3^ Pa. Every two dishes were placed sequentially in target chamber. The upper one was exposed to ion-beam bombardment, while the lower one was unexposed as a control. After ion bombardment, each plate was washed by 1 mL of sterile distilled water and diluted to the concentration of 10^3^ conidia/ml. The conidial suspension was spread on PDA medium and the plates were incubated at 25°C for 5 d. Conidia lethality rate was calculated as described for the UV mutagenesis.

For each single-agent mutagenesis, at least 50 single-colonies growing on PDA medium containing 1800 μg/mL propamocarb, which is the minimum inhibition concentration for *L*. *lecanii*, were picked out, single-spore isolated, numbered, transferred to PDA slants and stored at 4°C. Then 0.1 μL aliquots of conidial suspension (10^3^ conidia/ml) of the mutants and the wild-type isolate were inoculated in PDA medium containing 503.6 μg/mL propamocarb, which is the EC_50_ of the wild-type isolate (Xie et al., 2018a). Colony diameter was measured in five replicated plates on the fifth day after incubation. A positive mutant is defined as a mutant whose diameter of colony is 1-fold larger than that of the wild-type isolate. Positive mutation rates were calculated by the formula:positive mutation rate = number of positive mutants/number of colonies × 100%. Names of the highest propamocarb-tolerant mutants from each single-agent mutagenesis were shown in Table 1.

**Table 1:** Effects of mutagenesis modes and mutagenic agents on *L. lecanii* and names of the highest propamocarb-tolerant mutants produced in each mutagenesis mode.^a^

Mutagenesis modes	Mutagenic agents	Conidial lethality rates (%)	Positive mutation rates (%)	Mutant names
Single	NTG (N)	84.6 ± 8.1	7.7 ± 0.8 hij	Ll-N
	UV-light (U)	78.8 ± 6.2	7.7 ± 0.5 hij	Ll-U
	N^+^ ion-beam (I)	87.2 ± 9.1	11.4 ± 1.2 abcde	Ll-I
Consecutive (C)	N/U	85.5 ± 7.4	8.1 ± 0.7 fghij	Ll-C-NU
	U/N	85.3 ± 6.5	7.9 ± 0.4 ghij	Ll-C-UN
	N/I	90.6 ± 9.3	10.6 ± 0.9 abcdefgh	Ll-C-NI
	I/N	88.3 ± 9.1	11.1 ± 1.3 abcdef	Ll-C-IN
	U/I	88.9 ± 7.6	11.3 ± 1.2 abcde	Ll-C-UI
	I/U	89.9 ± 6.9	10.9 ± 0.8 abcdefg	Ll-C-IU
	N/U/I	92.6 ± 9.3	12.5 ± 1.5 abc	Ll-C-NUI
	N/I/U	90.8 ± 9.1	10.3 ± 1.2 bcdefghi	Ll-C-NIU
	U/N/I	92.9 ± 8.9	11.8 ± 0.9 abc	Ll-C-UNI
	U/I/N	93.1 ± 9.4	10.4 ± 1.2 abcdefghi	Ll-C-UIN
	I/N/U	90.5 ± 8.7	9.8 ± 0.8 cdefghij	Ll-C-INU
	I/U/N	91.9 ± 7.8	9.9 ± 1.1 cdefghij	Ll-C-IUN
Intermittent (I)	N + U	75.9 ± 8.1	7.4 ± 0.6 ij	Ll-I′-NU
	U + N	82.9 ± 7.9	7.6 ± 0.7 hij	Ll-I-UN
	N + I	88.9 ± 9.1	11.5 ± 1.1 abcde	Ll-I-NI
	I + N	85.4 ± 7.6	7.5 ± 0.5 hij	Ll-I-IN
	U + I	89.6 ± 9.6	10.6 ± 0.8 abcdefgh	Ll-I-UI
	I + U	81.5 ± 7.6	7.8 ± 0.6 ghij	Ll-I-IU
	N + U + I	90.6 ± 8.6	13.1 ± 1.4 ab	Ll-I-NUI
	N + I + U	82.6 ± 7.3	8.1 ± 0.6 fghij	Ll-I-NIU
	U + N + I	87.6 ± 8.4	11.6 ± 0.9 abcd	Ll-I-UNI
	U + I + N	84.9 ± 7.9	8.4 ± 0.7 efghij	Ll-I-UIN
	I + N + U	79.8 ± 8.1	7.8 ± 0.7 ghij	Ll-I-INU
	I + U + N	84.3 ± 8.4	8.6 ± 0.9 defghij	Ll-I-IUN
Consecutive+Intermittent (CI)	I/N/U + U	80.8 ± 7.5	7.1 ± 0.8 j	Ll-CI-U
	I/N/U + U + N	86.2 ± 8.2	7.6 ± 0.6 hij	Ll-CI-UN
	I/N/U + U + N + I	88.6 ± 8.7	13.5 ± 1.5 a	Ll-CI-UNI
Intermittent+Consecutive (IC)	U + N + I + I/N/U	91.2 ± 8.6	10.4 ± 0.9 abcdefghi	Ll-IC-UNI

a Results were mean ± SD (*n* = 3). Different lowercase letters following means in a column indicate significant differences among mutagenesis modes and mutagenic agents according to the LSD test (*P* < 0.05).

### Multiple-agent composite mutagenesis and mutant selection

Permutations of two-agent treatments (N and U, N and I, U, and I) and three-agent treatments (N, U, and I) were set with both consecutive and intermittent mode. Parameters for single-agent mutagenesis of the “N,” “U”, and “I” were also used in composite mutagenesis. Mutant selection was conducted only after treatment of the final agent in the consecutive mode and performed as described in single-agent mutagenesis. For the intermittent mode, the mutant selection was conducted after the treatment of each agent, and the highest propamocarb-tolerant mutant from former treatment was used as the starting isolate for later treatment. Names of the highest propamocarb-tolerant mutants from composite mutagenesis were also shown in Table 1. Contributive rates of each agent calculated by gradual factorization of the increment of EC_50_ value (ΔEC_50_) of each mutant in different mutagenesis modes were classified into N, U, I, and I/N/U (Table 2).

**Table 2:** Contributive rates of each mutagenic agent on the propamocarb-tolerance of mutants and co-improvement coefficients of mutagenic agents in composite mutagenesis mode.^a^

Mutagenesis modes	Mutants	ΔEC_50_ (μg/mL)	Contributive rates (%)				CIC
			N	U	I	I/N/U	
“N”	Ll-N	546.4 ± 9.2	–	–	–	–	–
“U”	Ll-U	65.4 ± 2.2	–	–	–	–	–
“I”	Ll-I	720 ± 21.2	–	–	–	–	–
“I/N/U”	Ll-C-INU	1,011 ± 48.7	–	–	–	–	–
“N + U”	Ll-I-NU	809 ± 32.7	67.5 ± 1.6	32.5 ± 1.6	–	–	132.2 ± 2.9 ab
“U + N”	Ll-I-UN	837.9 ± 28.2	92.2 ± 0.0	7.8 ± 0.0	–	–	137.0 ± 2.1 a
“N + I”	Ll-I-NI	1139.9 ± 35.4	47.9 ± 0.7	–	52.1 ± 0.7	–	90.0 ± 0.6 ij
“I + N”	Ll-I-IN	1182.3 ± 34.5	39.1 ± 0.0	–	60.9 ± 0.0	–	93.4 ± 0.5 hi
“U + I”	Ll-I-UI	954.4 ± 25.0	–	6.9 ± 0.1	93.1 ± 0.1	–	121.5 ± 0.4 c
“I + U”	Ll-I-IU	909.2 ± 21.6	–	20.8 ± 0.5	79.2 ± 0.5	–	115.8 ± 0.7 d
“N + U + I”	Ll-I-NUI	1,450 ± 49.0	37.7 ± 0.6	18.1 ± 1.0	44.2 ± 0.4	–	108.9 ± 1.0 f
“N + I + U”	Ll-I-NIU	1,299 ± 45.8	42.1 ± 0.8	12.2 ± 0.4	45.7 ± 0.4	–	97.5 ± 1.1 gh
“U + N + I”	Ll-I-UNI	1622.9 ± 55.6	47.6 ± 0.0	4.0 ± 0.0	48.4 ± 0.0	–	121.9 ± 1.2 c
“U + I + N”	Ll-I-UIN	1584.8 ± 55.0	39.8 ± 0.5	4.1 ± 0.0	56.1 ± 0.5	–	119.0 ± 1.2 cd
“I + N + U”	Ll-I-INU	1,471 ± 52.5	31.4 ± 0.2	19.6 ± 0.5	48.9 ± 0.3	–	110.5 ± 1.2 ef
“I + U + N”	Ll-I-IUN	1411.9 ± 45.6	35.6 ± 0.6	13.4 ± 0.4	51.0 ± 0.1	–	106.0 ± 0.8 f
“I/N/U + U”	Ll-CI-U	1234.7 ± 37.5	–	18.1 ± 1.5	–	81.9 ± 1.5	114.7 ± 1.9 de
“I/N/U + U + N”	Ll-CI-UN	1624.8 ± 64.5	24.0 ± 0.7	13.8 ± 1.2	–	62.2 ± 0.5	100.1 ± 0.3 gh
“I/N/U + U + N + I”	Ll-CI-UNI	2055.1 ± 132.0	19.0 ± 0.1	10.9 ± 1.3	20.9 ± 1.9	49.2 ± 0.8	87.7 ± 2.6 j
“U + N + I + I/N/U”	Ll-IC-UNI	3072.8 ± 185.8	25.1 ± 0.7	2.1 ± 0.1	25.5 ± 0.7	47.2 ± 1.4	131.2 ± 3.4 b

^a^Results were mean ± SD (*n* = 3). The ΔEC_50_ referred to the increment of the median effective concentration. The EC_50_ value of the wild-type isolate as untreated control was 503.6 ± 15.9 μg/mL propamocarb. Contributive rates of each agent in a composite mutagenesis mode were calculated by gradual factorization of the increment of EC_50_ value of each mutant. For an example of the Ll-I-NU, the first increment of EC_50_ value from N was 546.4 μg/mL, and then the second increment of EC_50_ value from U was 262.6 μg/mL. The contributive rate of N and U in the “N + U” mutagenesis mode equaled 809 divided by 546.4 and 262.6, respectively. The CIC referred to the co-improvement coefficient. Different lowercase letters following means in a column indicates significant differences among mutagenesis modes according to the LSD test (*P* < 0.05).

To determine whether synergistic effects exist among the different agents in a composite mutagenesis, the theoretical increment of EC_50_ value of each mutant was calculated by adding up the increment of EC_50_ value (ΔEC_50_) from each agent in a certain mutagenesis. Lacking of a proper evaluation method on synergistic effects of a composite mutagenesis, the co-improvement coefficient (CIC) was introduced according to the co-toxicity coefficient often used in the assessment of compound pesticides (Sun and Johnson, 1960). The CIC for a composite mutagenesis was calculated according to the following: if CIC > 120, it shows a synergistic effect, whereas CIC < 80 indicates an antagonistic effect, and CIC between 80 and 120 is considered as an additive effect. If a composite mutagenesis (CM) involves i agents (A1, A2, A3, … and Ai), and all agents have ΔEC_50_, then the following formulas were used (A1 serving as the standard):

Improvement index (II) of A1 = 100.

Improvement index (II) of Ai = (ΔEC_50_ of Ai / ΔEC_50_ of A1) × 100.

Actual improvement index (AII) of CM = (ΔEC_50_ of CM / ΔEC_50_ of A1) × 100.

Theoretical improvement index (TII) of CM = II of A1 + II of A2 + … + II of Ai.

Co-improvement coefficient (CIC) = AII of CM / TII of CM × 100.

### Mitotic stability and propamocarb sensitivity

All the highest propamocarb-tolerant mutants from each single- and multiple-agent mutagenesis were chosen. Their mitotic stability was tested, respectively, by 5, 10, 15, and 20 passages on PDA medium and then by a further passage on PDA medium supplemented with 1800 μg/mL propamocarb. For sensitivity to propamocarb, 100 μL aliquots of conidial suspension (10^3^ conidia/ml) of each mutant or the wild-type isolate were uniformly spread on WA media (20 g agar per liter of distilled water) supplemented with different propamocarb concentrations of 0, 240, 480, 960, 1920, 3,840, and 7,680 μg/mL, respectively. Each concentration was replicated for five plates. All the plates were incubated at 25°C for 16 h, and then conidial germination rates were recorded under a light microscope (Olympus, Olympus Corporation, Tokyo, Japan) with a magnification of 400. A germinated spore is defined as one whose gem tube is longer than half of the spore width (Leland et al., 2005). The median effective concentration (EC_50_) value for each isolate was calculated by regressing percentage of germination inhibition against log value of each fungicide concentration as described previously (Zhai et al., 2013). The tolerance ratio (TR) was calculated by dividing the EC_50_ value of each positive mutant by that of the wild-type isolate (Fitriana et al., 2015). All the tests were performed three times.

### Colony growth, conidial yield and germination on plates

Colony growth, conidial yield and germination on PDA plates were tested for all the highest propamocarb-tolerant mutants as described previously (Zhang et al., 2016). To determine colony growth, a drop of 1 μL conidial suspension (10^3^ conidia/ml) was inoculated in the middle of PDA plate. Diameters of the colonies were measured after 7 d cultivation at 25°C. To determine conidial yield, 200 μL of conidial suspension (10^6^ conidia/ml) were equally plated on PDA plates. An agar disk of 8 mm in diameter was randomly taken from each plate at 14 d and put into 10 mL of 0.05% (v/v) Tween-80 solution. Conidial yield was reported by number of conidia per mm^2^. To determine germination rate, 50 μL of conidial suspension (10^5^ conidia/ml) were equally plated on WA plates. Conidial germination rate was recorded after 16 h cultivation at 25°C. Each treatment had five replicates and all the tests were performed three times.

### Virulence and sporulation on aphid cadavers

Virulence of all the highest propamocarb-tolerant mutants and the wild-type isolate were assayed with adult cotton aphids (*Aphis gossypii* Glover) by using an immersion method (Hall, 1984). Aphids were placed in a net, immersed for 10 s in the conidial suspension of five different concentrations (10^4^, 10^5^, 10^6^, 10^7^, and 10^8^ conidia/ml), laid on sterilized filter paper to draw off surplus suspension and then fed on potted cucumber plants. Control aphids were treated with 0.05% (v/v) Tween-80 solution only. Each treatment had five replicates with 200 aphids per replicate. The experiment was repeated three times. Aphid mortality was recorded every 12 h and cadavers were transferred to moisturized filter paper (Double Ring, Hangzhou Whatman-Xinhua Filter Paper Co., Ltd., Hangzhou, China) to monitor emergence of the fungal hyphae. The median lethal concentration (LC_50_) value and the median survival time (LT_50_) value were calculated as described previously (Zhang et al., 2016). To determine sporulation on aphid cadavers, the dead aphids inoculated with the mutants or the wild-type isolate at the concentration of 10^7^ conidia/ml were collected, weighed and maintained at 25°C and in high humidity of above 85% for 7 d. Twenty sporulated aphids were cut into very small pieces with a sterile knife and added to a tube of 5 mL sterile water containing 0.05% Tween-80 solution. The mixture was then stirred for 2 h before the conidia were counted with an improved Neubauer chamber (Hausser Scientific, PA, United States) under a light microscope (Olympus, Olympus Corporation, Tokyo, Japan) with a magnification of 400. Sporulation on aphid cadavers was reported by number of conidia per gram of dead aphids.

### Cultural growth and cell membrane permeability

Mycelium disks 5 mm in diameter from 5-day-old colonies of the 7 mutants (Ll-N, Ll-U, Ll-I, Ll-C-INU, Ll-I-UNI, Ll-CI-UNI, and Ll-IC-UNI) and the wild-type isolate grown on propamocarb-free PDA medium were transferred into 250 mL flasks (10 disks per flask) (SHUNIU, Sichuan Shubo (Group) Co., Ltd., Chongzhou, China) containing 150 mL of Potato Dextrose Broth (PDB) liquid medium (200 g potato and 20 g glucose per liter of distilled water) with or without propamocarb. The flasks were shaken at 175 rpm and 25°C for 7 d and the mycelia were harvested every day on double gauze and washed twice with double-distilled water. Fungal biomass in liquid medium was measured by weighing the mycelia after freeze drying at −40°C for 48 h.

Mycelium mat collected on the fourth day were filtrated in vacuum for 20 min, and 0.5 g of sample were suspended in 25 mL of ultrapure water. After the incubation for 0, 5, 10, 20, 40, 60, 80, 100, 120, 140, 160, and 180 min, an electrical conductivity meter (CON510-Eutech/Oakton, Thermofisher Scientific, Singapore) was used to assess the extent of leaching of cell contents through cell membranes (Duan et al., 2013). Mycelia incubated for 180 min were boiled for 5 min, and the final conductivity was measured. Cell membrane permeability was reflected by relative conductivity of mycelia which was calculated as:relative conductivity = conductivity at different times/final conductivity ×100%. Each treatment had five replicates, and the experiment was performed three times.

### Lipid extraction and analysis

Mycelia of the above 7 mutants and the wild-type isolate were harvested after 4 d incubation in PDB liquid medium with or without propamocarb, and the resulting mycelial mat were washed twice with double-distilled water. 0.5 g of fresh tissue was extracted for lipid by the method of Cartwright (1993). Total lipid extracts were separated into lipid classes by thin-layer chromatography (TLC) on silica gel G60 plates (Merck, Darmstadt, Germany) (Kates, 1986). Polar lipids were separated by two-dimension TLC using chloroform/methanol/acetic acid/water [170:30:20:7 (v/v)] and chloroform/methanol/ammonium hydroxide [60:25:4 (v/v)], allowing phosphatidic acid (PtdOH) to be resolved from other phosphoglycerides. Neutral lipids were separated using petroleum ether/diethyl ether/acetic acid [80:20:1 (v/v)]. The lipid bands separated by TLC were routinely visualized by spraying the plates with 8-anilino-1-napthalenesulphonic acid (ANSA) in anhydrous methanol [0.2% (w/v)], and viewing under UV light. Routine identification was by comparison with standards, but further identification was made by using color stains and destructive reagents as described in Kates (1986). All of chemical agents used in lipid analysis were purchased form Sigma-Aldrich, Shanghai.

### Control against aphids

Bioassays were conducted to test ability of the above 7 mutants to work as a suitable biocontrol agent under application of propamocarb as described previously (Zhang et al., 2014; Xie et al., 2018a). Potted cucumber plants were grown at 24 ± 1°C and 16 L:8D for 5 weeks until the four-leaf stage in a controlled environmental chamber (Changzhou Nuoji Instrument Co., Ltd., Changzhou, China). Twenty newly-grown synchronized cotton aphid adults, as described above, were then transferred to each cucumber plant with a soft hair brush and allowed to settle on the plant for 6 h. Propamocarb solution was sprayed at the field recommended dose of 550 μg/mL on foliage of potted cucumber plants from both sides until runoff using a Research Track Sprayer (DeVries Manufacturing Hollandale, MN, United States). The cucumber plants sprayed by the same volume of water were as the control. On the day 0 and 3 after spraying propamocarb or water, conidial suspension (10^5^ conidia/mL) plus water control were applied to potted cucumber plants using the Research Track Sprayer as described above, calibrated to deliver 0.10 ± 0.02 mL/cm^2^. Each treatment had three replicates with nine potted cucumber plants per replicate. After treatment, the plants were air-dried at room temperature for 1 h. The plants in each replicate of each treatment were then individually covered in a fabric sleeve to prevent aphid escape and placed in cages (50 × 50 × 50 cm). All the cages in this trail were arrayed in a completely randomized design and maintained at 24 ± 1°C, 85 ± 2% RH and 16 L:8D in a controlled environment chamber. The plants were watered every 2 days and fertilized every 7 days with N-P-K (20–20-20) at 200 μg/mL. Aphid mortality was recorded every 12 h until the 7th day after fungal inoculation. The bioassay was performed three times.

### Data analysis

Data were subjected to one-way analyses of variance (ANOVA) to determine the effects of mutagenic agents on contributive rates of in the repeated test of different degree, and to determine the effects of different mutagenesis modes on conidial lethality rates, positive mutation rates and CIC, and to determine the changes of mutants in fungal propamocarb sensitivity, mitotic stability, mycelial growth speed on plates or in liquid, sporulation on plates or aphids, conidial germination, LC_50_ and LT_50_ to aphids, lipid constituent, cell membrane permeability, and control efficacy against aphids in the repeated test of same degree. Data were analyzed for homogeneity of variance and normality using the Leven and Shapiro–Wilk tests before statistical analyses. Significant differences were analyzed by the Fisher’s least significant difference (LSD) test when the F-tests were statistically significant at *p* < 0.05. All statistical analyses were performed with the SPSS 19.0 (SPSS Inc., Chicago, United States).

## Results

### Effects of mutagenesis modes and agents

**Figure 1:**
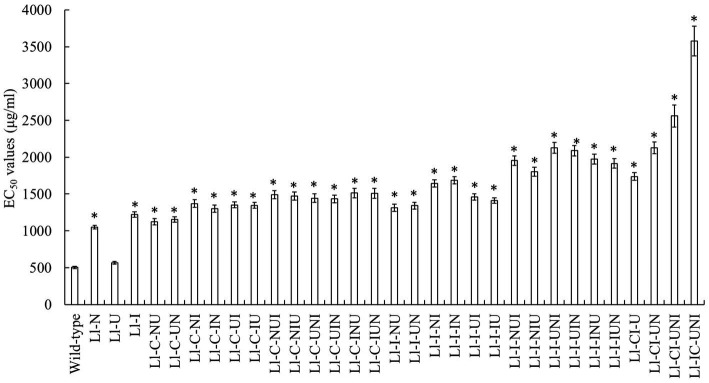
Propamocarb sensitivity of *L. lecanii* isolates including the highest-tolerant mutants from each single and composite mutagenesis. Data were means ± SD (*n* = 3). The EC_50_ referred to the median effective concentration. Significances between means for each mutant and the wild-type isolate were shown as *p < 0.05 according to the LSD test.

Conidial lethality rates were not significantly different in various mutagenesis modes (*F* = 0.873, df = 62, *p* = 0.652), ranging from 75.9 to 93.1% (Table 1). There were significant differences in positive mutation rates from different mutagenesis modes (*F* = 12.24, df = 62, *p* < 0.001), with a range of 7.1 to 13.5% (Table 1). The highest positive mutation rate of 13.5% occurred in the intermittent three-agent treatment of UV-light, NTG and N^+^ ion-beam after the consecutive three-agent treatment of N^+^ ion-beam, NTG and UV-light (“I/N/U + U + N + I”). However the consecutive three-agent treatment of N^+^ ion-beam, NTG and UV-light after the intermittent three-agent treatment of UV-light, NTG and N^+^ ion-beam (“U + N + I + I/N/U”) produced the highest propamocarb-tolerant mutant Ll-IC-UNI having a TR of 7.10. Other mutants, with the exception of the Ll-U, also had significantly higher TR ranging from 2.08 to 5.08 (*F* = 206.40, df = 64, *p* < 0.001) (Figure 1). Furthermore, the contributive rate of I/N/U (60.1%) was the largest followed by I (52.2%), N (42.2%) and U (13.2%), and the contributive rates of I/N/U and I were significantly higher than those of N and U (*F* = 52.16, df = 125, *p* < 0.001) (Table 2). Through calculation of CTC, synergistic effects were found in the “N + U,” “U + N,” “U + I,” U + N + I” and “U + N + I + I/N/U,” and CTC in the “U + N + I + I/N/U was significantly higher than CTC in other composite mutagenesis modes excepting the “N + U” and “U + N (*F* = 257.6, df = 32, *p* < 0.001) (Table 2).

### Mitotic stability and biological characteristics

Total 32 isolates including all the highest propamocarb-tolerant mutants in each single- and composite-mutagenesis and the wild-type isolate, were multi-generated to assess mitotic stabilities of their propamocarb-tolerance (Table 3). EC_50_ values significantly increased on the fifth-generation and then declined in 6 mutants that were produced in single- and composite-mutagenesis using N^+^ ion-beam (Ll-I:*F* = 5.31, df = 10, *p* = 0.015; Ll-C-NI:*F* = 3.82, df = 10, *p* = 0.038; Ll-C-NUI:*F* = 3.53, df = 10, *p* = 0.048; Ll-C-UNI:*F* = 5.32, df = 10, *p* = 0.015; Ll-I-NI:F_0_ = 4.62, df = 10, *p* = 0.023; Ll-I-UI:*F* = 4.06, df = 10, *p* = 0.033). However, all the EC_50_ values of these 31 mutants kept un-decreased during 20 passages. Biological characteristics of the above isolates were shown in Table 4. Compared to the wild-type isolate, only 4 mutants (Ll-C-NU, Ll-C-UNI, Ll-I-NU, and Ll-I-INU) showed significant differences in colony growth on plates (*F* = 9.89, df = 64, *p* < 0.001), sporulation on plates (*F* = 5.71, df = 64, *p* < 0.001) and sporulation on aphids (*F* = 11.96, df = 64, *p* < 0.001), and LC_50_ to aphids (*F*_4_ = 3.125, df = 64, *p* < 0.001).

**Table 3:** Mitotic stability of propamocarb sensitivity for the *L. lecanii* isolates or mutants.^a^

Isolates/mutants	0th generation	5th generation	10th generation	15th generation	20th generation
Wild-type	503.6 ± 15.9	512.5 ± 16.8	509.4 ± 15.3	511.3 ± 14.2	502.1 ± 15.7
Ll-N	1050.0 ± 25.1	1046.8 ± 33.2	1052.7 ± 36.4	1063.4 ± 30.2	1058.1 ± 34.1
Ll-U	569.0 ± 18.1	571.3 ± 15.4	584.2 ± 18.3	568.4 ± 21.4	566.2 ± 17.2
Ll-I	1223.6 ± 37.1	1341.6 ± 40.2^* b^	1284.3 ± 35.6	1251.6 ± 34.1	1224.9 ± 38.1
Ll-C-NU	1123.3 ± 40.2	1130.4 ± 32.1	1128.36 ± 41.2	1120.4 ± 32.1	1127.1 ± 30.7
Ll-C-UN	1154.2 ± 35.6	1142.9 ± 30.9	1149.3 ± 31.5	1150.1 ± 29.4	1150.9 ± 34.7
Ll-C-NI	1369.7 ± 52.6	1489.4 ± 39.7^*^	1436.8 ± 42.6	1389.4 ± 45.9	1377.2 ± 40.8
Ll-C-IN	1302.4 ± 50.4	1408.6 ± 40.6	1372.5 ± 39.7	1338.4 ± 41.5	1311.3 ± 44.9
Ll-C-UI	1352.6 ± 41.7	1450.3 ± 42.7	1416.3 ± 45.6	1366.4 ± 40.9	1350.1 ± 35.8
Ll-C-IU	1346.5 ± 40.6	1450.2 ± 39.7	1393.2 ± 36.5	1358.4 ± 39.4	1341.3 ± 41.2
Ll-C-NUI	1489.7 ± 55.3	1613.2 ± 48.4^*^	1553.2 ± 52.3	1498.4 ± 46.9	1485.1 ± 50.1
Ll-C-NIU	1472.4 ± 56.7	1577.1 ± 42.3	1501.6 ± 49.6	1470.1 ± 52.3	1476.3 ± 55.4
Ll-C-UNI	1442.3 ± 57.9	1581.3 ± 40.1^*^	1498.3 ± 52.1	1423.6 ± 48.6	1436.8 ± 44.2
Ll-C-UIN	1432.6 ± 51.6	1544.2 ± 58.1	1501.7 ± 42.9	1459.7 ± 4,732	1441.8 ± 50.3
Ll-C-INU	1514.6 ± 64.6	1622.5 ± 49.4	1577.1 ± 58.3	1521.4 ± 48.3	1517.9 ± 51.7
Ll-C-IUN	1510.3 ± 67.4	1630.6 ± 44.8	1594.1 ± 52.7	1551.0 ± 60.1	1504.6 ± 59.4
Ll-I-NU	1312.6 ± 48.6	1321.4 ± 39.1	1315.8 ± 45.6	1310.8 ± 38.7	1315.9 ± 42.7
Ll-I-UN	1341.5 ± 44.1	1340.2 ± 32.1	1352.7 ± 35.1	1354.1 ± 30.8	1350.7 ± 42.1
Ll-I-NI	1643.5 ± 51.3	1788.6 ± 50.3^*^	1705.2 ± 42.9	1656.4 ± 39.4	1659.7 ± 54.3
Ll-I-IN	1685.9 ± 50.4	1694.2 ± 51.9	1701.3 ± 48.9	1688.1 ± 44.3	1694.1 ± 55.8
Ll-I-UI	1458.9 ± 40.9	1566.3 ± 45.8^*^	1519.4 ± 32.1	1476.2 ± 30.8	1460.7 ± 45.9
Ll-I-IU	1412.8 ± 37.5	1412.6 ± 40.8	1426.4 ± 45.2	1421.0 ± 32.9	1418.4 ± 38.7
Ll-I-NUI	1953.6 ± 64.9	2059.6 ± 60.8	2004.7 ± 52.3	1975.9 ± 64.2	1960.1 ± 68.1
Ll-I-NIU	1802.6 ± 61.7	1805.6 ± 61.3	1819.4 ± 58.3	1821.3 ± 61.0	1817.4 ± 60.3
Ll-I-UNI	2126.5 ± 71.5	2289.4 ± 63.1	2214.3 ± 59.7	2155.1 ± 65.7	2131.4 ± 69.1
Ll-I-UIN	2088.4 ± 70.9	2098.2 ± 58.4	2091.3 ± 65.9	2087.1 ± 67.1	2080.3 ± 56.3
Ll-I-INU	1974.6 ± 68.4	1979.3 ± 55.2	1974.1 ± 61.3	1970.2 ± 52.3	1969.8 ± 50.1
Ll-I-IUN	1915.5 ± 61.5	1925.6 ± 51.3	1921.0 ± 49.6	1919.8 ± 56.4	1917.4 ± 62.3
Ll-CI-U	1738.3 ± 53.4	1748.3 ± 50.4	1744.3 ± 63.1	1758.1 ± 61.4	1730.3 ± 59.0
Ll-CI-UN	2128.4 ± 80.4	2138.4 ± 65.4	2153.4 ± 73.4	2108.5 ± 69.2	2155.4 ± 73.6
Ll-CI-UNI	2558.7 ± 147.9	2769.8 ± 174.9	2622.3 ± 159.6	2581.3 ± 140.3	2574.8 ± 139.4
Ll-IC-UNI	3576.4 ± 201.7	3899.4 ± 178.6	3776.3 ± 184.3	3625.7 ± 176.9	3587.1 ± 165.3

a Results were the median effective concentration (EC_50_) values (μg/mL) and shown with means ± SD (*n* = 3).

^b^Significances between means of the generations for each isolate in a row were shown as ^*^P < 0.05 according to the LSD test.

**Table 4:** Growth characteristics and virulence of the mutants and the wild-type isolate of *L. lecanii.*^a^

Isolates/mutants	Colony growth on plates (mm)	Sporulation on plates (×10^6^ conidia/mm^2^)	Sporulation on aphids (×10^9^ conidia/g) ^b^	Conidial germination (%)	LC_50_ to aphids (×10^6^ conidia/ml)	LT_50_ to aphids (d)^c^
Wild-type	22.4 ± 0.5	2.4 ± 0.3	3.5 ± 0.3	98.5 ± 3.2	1.7 ± 0.2	3.5 ± 0.3
Ll-N	22.4 ± 0.5	2.3 ± 0.2	3.5 ± 0.4	97.5 ± 3.5	1.7 ± 0.3	3.5 ± 0.3
Ll-U	22.3 ± 0.2	2.4 ± 0.2	3.2 ± 0.3	98.1 ± 3.1	1.7 ± 0.2	3.5 ± 0.4
Ll-I	22.5 ± 0.4	2.4 ± 0.2	3.4 ± 0.2	98.2 ± 3.2	1.7 ± 0.2	3.5 ± 0.2
Ll-C-NU	22.3 ± 0.2	2.5 ± 0.3	2.1 ± 0.3 ^*^	98.1 ± 3.3	1.2 ± 0.2	3.5 ± 0.4
Ll-C-UN	22.5 ± 0.5	2.2 ± 0.2	3.5 ± 0.1	97.5 ± 3.7	1.8 ± 0.2	3.5 ± 0.4
Ll-C-NI	22.2 ± 0.2	2.4 ± 0.2	3.2 ± 0.3	98.0 ± 3.2	1.7 ± 0.3	3.5 ± 0.3
Ll-C-IN	22.6 ± 0.3	2.0 ± 0.1	3.5 ± 0.5	98.8 ± 3.0	1.7 ± 0.2	3.5 ± 0.4
Ll-C-UI	22.1 ± 0.2	2.4 ± 0.2	3.1 ± 0.3	97.8 ± 3.2	1.9 ± 0.4	3.5 ± 0.3
Ll-C-IU	22.3 ± 0.5	2.3 ± 0.3	3.3 ± 0.3	98.5 ± 3.2	1.7 ± 0.2	3.5 ± 0.4
Ll-C-NUI	22.4 ± 0.3	2.3 ± 0.2	3.5 ± 0.4	98.2 ± 3.4	1.6 ± 0.3	3.5 ± 0.4
Ll-C-NIU	22.6 ± 0.3	2.4 ± 0.2	3.4 ± 0.3	98.4 ± 3.1	1.7 ± 0.2	3.5 ± 0.3
Ll-C-UNI	18.5 ± 0.2^*,^^d^	3.4 ± 0.2^*^	3.3 ± 0.2	98.5 ± 3.2	2.4 ± 0.2	3.5 ± 0.3
Ll-C-UIN	22.4 ± 0.4	2.4 ± 0.2	3.5 ± 0.3	98.3 ± 3.2	1.7 ± 0.2	3.5 ± 0.4
Ll-C-INU	22.5 ± 0.5	2.2 ± 0.3	3.5 ± 0.3	98.5 ± 3.4	1.7 ± 0.3	3.5 ± 0.2
Ll-C-IUN	22.7 ± 0.5	2.2 ± 0.2	3.1 ± 0.3	98.6 ± 3.4	1.8 ± 0.2	3.5 ± 0.4
Ll-I-NU	22.3 ± 0.5	1.1 ± 0.1^*^	0.5 ± 0.1^*^	97.9 ± 3.2	1.7 ± 0.2	3.0 ± 0.2
Ll-I-UN	22.4 ± 0.3	2.4 ± 0.2	3.2 ± 0.3	98.1 ± 3.2	1.6 ± 0.3	3.5 ± 0.4
Ll-I-NI	22.5 ± 0.4	2.3 ± 0.1	3.5 ± 0.4	98.5 ± 3.3	1.7 ± 0.3	3.5 ± 0.2
Ll-I-IN	22.5 ± 0.5	2.4 ± 0.2	3.3 ± 0.4	98.2 ± 3.2	1.6 ± 0.2	3.5 ± 0.3
Ll-I-UI	22.1 ± 0.5	2.3 ± 0.3	3.3 ± 0.5	98.3 ± 3.1	1.7 ± 0.2	3.5 ± 0.2
Ll-I-IU	22.3 ± 0.2	2.5 ± 0.3	3.1 ± 0.3	98.5 ± 3.1	1.7 ± 0.4	3.5 ± 0.4
Ll-I-NUI	22.4 ± 0.3	2.4 ± 0.2	3.6 ± 0.3	98.4 ± 3.4	1.8 ± 0.2	3.5 ± 0.4
Ll-I-NIU	22.7 ± 0.2	2.2 ± 0.3	3.5 ± 0.2	96.9 ± 3.0	1.7 ± 0.2	3.5 ± 0.5
Ll-I-UNI	22.1 ± 0.4	2.4 ± 0.2	3.2 ± 0.3	98.1 ± 3.2	1.7 ± 0.1	3.5 ± 0.4
Ll-I-UIN	22.3 ± 0.5	2.6 ± 0.4	3.3 ± 0.2	98.0 ± 3.2	1.6 ± 0.2	3.5 ± 0.4
Ll-I-INU	22.0 ± 0.2	2.4 ± 0.2	5.5 ± 0.6 ^*^	98.2 ± 3.5	2.9 ± 0.5 ^*^	4.5 ± 0.3
Ll-I-IUN	22.2 ± 0.5	2.3 ± 0.1	3.1 ± 0.3	98.5 ± 3.4	1.8 ± 0.3	3.5 ± 0.5
Ll-CI-U	22.3 ± 0.4	2.3 ± 0.2	3.3 ± 0.4	97.5 ± 2.5	1.7 ± 0.4	3.5 ± 0.3
Ll-CI-UN	22.2 ± 0.3	2.5 ± 0.3	3.2 ± 0.2	98.0 ± 4.4	1.8 ± 0.2	3.6 ± 0.4
Ll-CI-UNI	22.4 ± 0.4	2.5 ± 0.2	3.5 ± 0.5	98.6 ± 3.6	1.7 ± 0.2	3.5 ± 0.4
Ll-IC-UNI	22.5 ± 0.5	2.5 ± 0.2	3.3 ± 0.6	98.8 ± 3.2	1.6 ± 0.3	3.4 ± 0.3

^a^Results were means ± SD (*n* = 3). The LC_50_ referred to 50% lethal concentration and the LT_50_ refers to 50% lethal time.

^b^Aphids were inoculated with the concentration of 1 × 10^7^ conidia/mL by immersing.

c Aphids were inoculated with the concentration of 2 × 10^6^ conidia/mL by immersing.

d Significances between means for each mutant and the wild-type isolate in a column were shown ^*^P < 0.05 according to the LSD test.

### Mycelial growth and cell membrane permeability

**Figure 2:**
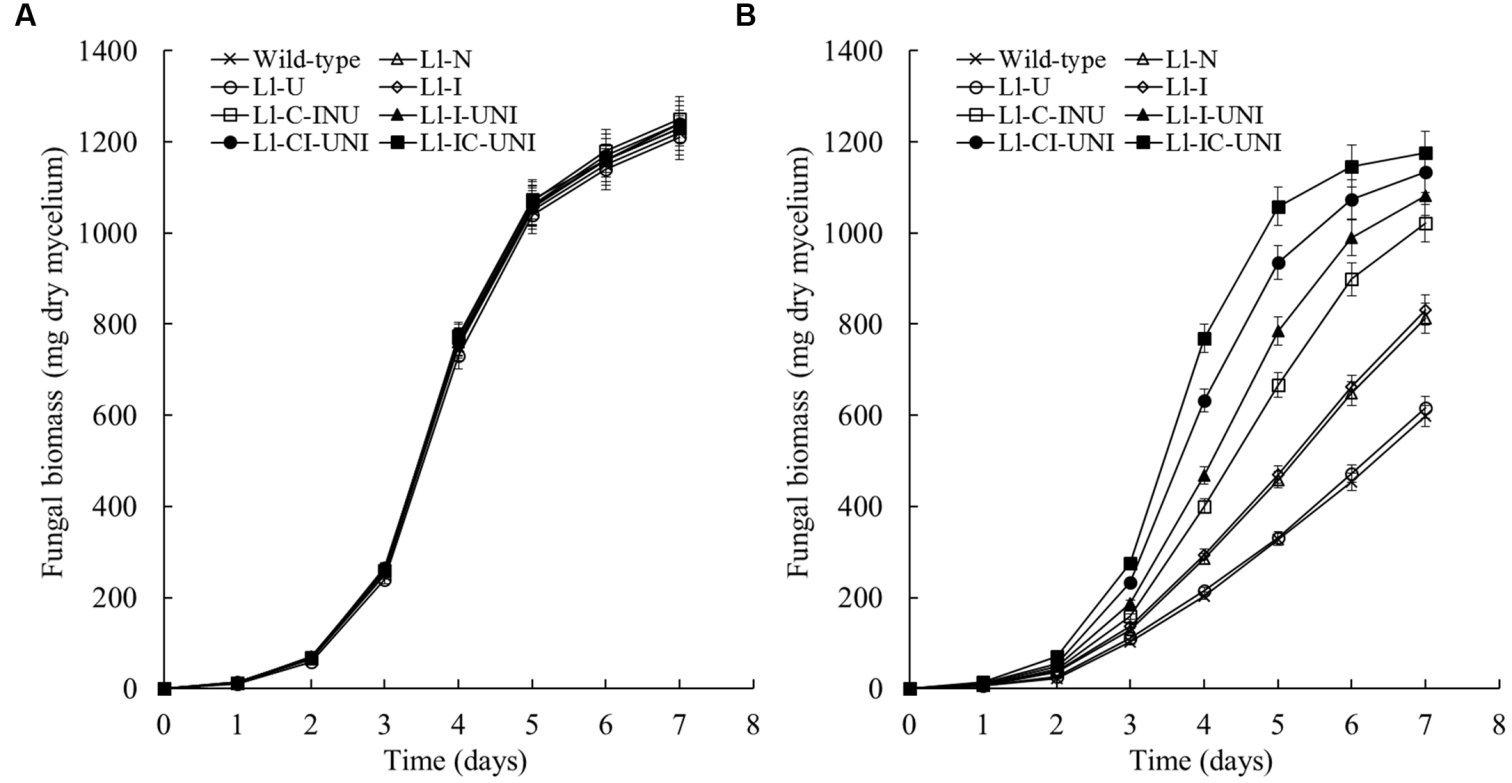
Fungal biomass of the 7 mutants and the wild-type isolate of *L. lecanii.* Cultures grew in PDB liquid medium **(A)** without and **(B)** with 550 μg/mL propamocarb. Data were means ± SD (*n* = 3).

Mycelial growth in PDB liquid medium without propamocarb was not significantly different between the 7 mutants (Ll-N, Ll-U, Ll-I, Ll-C-INU, Ll-I-UNI, Ll-CI-UNI, and Ll-IC-UNI) and the wild-type isolate of *L*. *lecanii* throughout the experimental period (*F* = 1.33, df = 128, *p* = 0.243) (Figure 2A). Field recommended dose of propamocarb (550 μg/mL) caused significant inhibition on mycelial growth of *L*. *lecanii* isolates (Figure 2B). Inhibition of mycelial growth with propamocarb were significantly different between the 7 mutants and the wild-type isolate from the 1st day (*F* = 159.00, df = 16, *p* < 0.001). The Ll-U was severely inhibited by propamocarb as with the wild-type isolate. Inhibition of the mycelial growth of other 6 mutants was less than the wild-type isolate to different extent. The Ll-IC-UNI was the least, which was followed by the Ll-CI-UNI, Ll-I-UNI, Ll-C-INU, Ll-I, and Ll-N. On the 7st day, inhibition percentage of the mycelial growth rate was 58.7% for the wild-type isolate, 57.3, 42.7, 41.3, 23.5, 16.4, 9.4, and 3.3% for the Ll-U, Ll-N, Ll-I, Ll-C-INU, Ll-I-UNI, Ll-CI-UNI, and Ll-IC-UNI, respectively.

**Figure 3:**
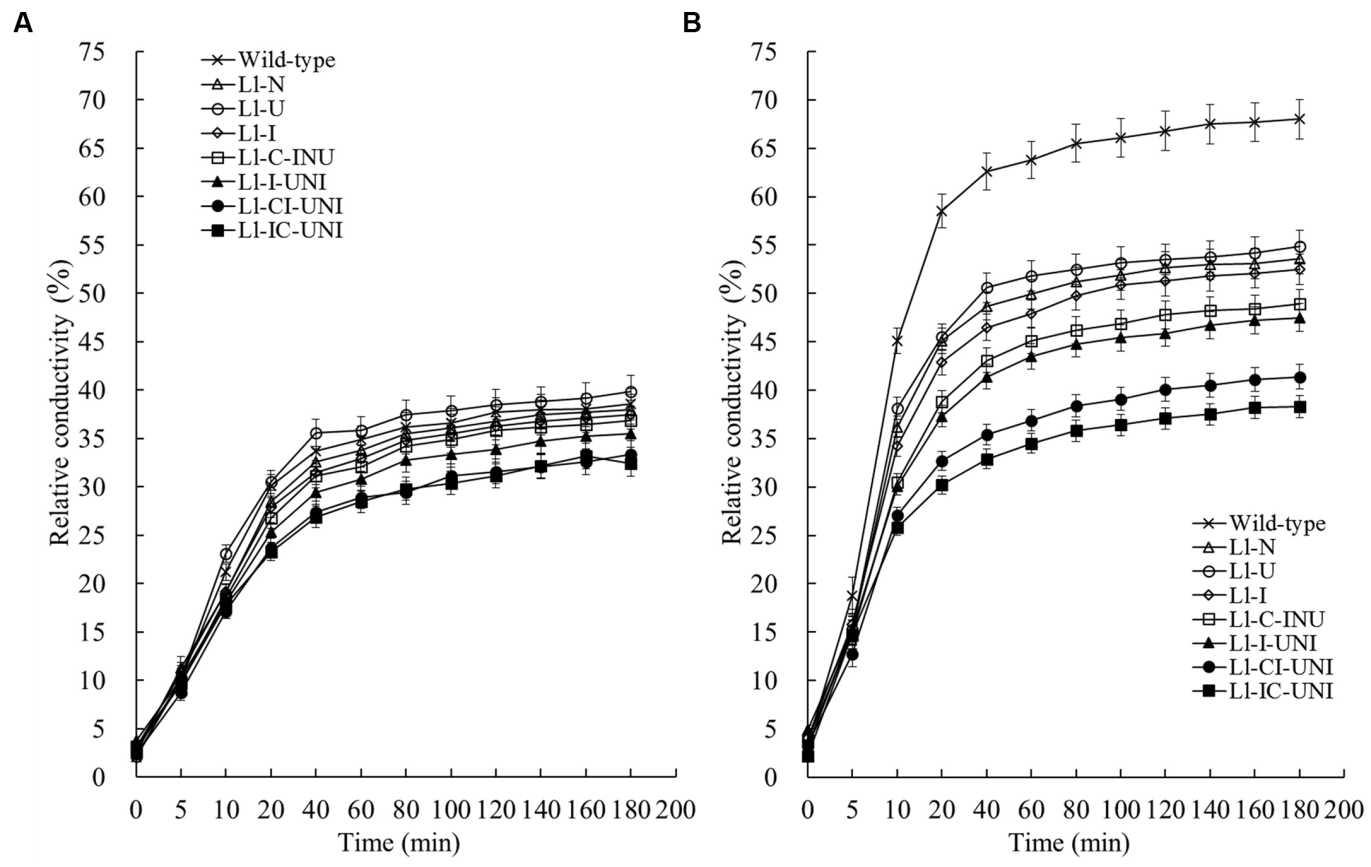
Cell membrane permeability as indicated by relative conductivity of the 7 mutants and the wild-type isolate of *L. lecanii*. Cultures grew in PDB liquid medium **(A)** without and **(B)** with 550 μg/mL propamocarb. Data were means ± SD (*n* = 3).

As indicated by relative conductivity, cell membrane permeability of *L*. *lecanii* isolates, whose mycelia were pre-incubated with or without 550 μg/mL propamocarb for 4 d, increased rapidly within 20 min and kept stable from 40 up to 180 min (Figures 3A,B). Without propamocarb (Figure 3A), cell membrane permeability was slightly higher in the Ll-U than that of the wild-type isolate at 180 min after incubation, but it was lower slightly in 3 mutants (Ll-N, Ll-I, and Ll-C-INU), moderately in the Ll-I-UNI, strongly and significantly in the other 2 mutants (Ll-CI-UNI, and Ll-IC-UNI) (*F* = 9.35, df = 16, *p* < 0.001). Incubation with propamocarb increased cell membrane permeability of all the isolates (Figure 3B). Compared to 76.2% of the wild-type isolate, the increase in cell membrane permeability of all the mutants was significantly less at 180 min after incubation (*F* = 466.7, df = 16, *p* < 0.001). Increase of cell membrane permeability of the Ll-IC-UNI was the least, which was increased by 15.1% and followed by 24.0, 32.5, 33.8, 37.6, 40.0, and 41.1% in the Ll-CI-UNI, Ll-I-UNI, Ll-C-INU, Ll-I, Ll-N, and Ll-U, respectively.

### Pattern and amount of lipids

**Figure 4:**
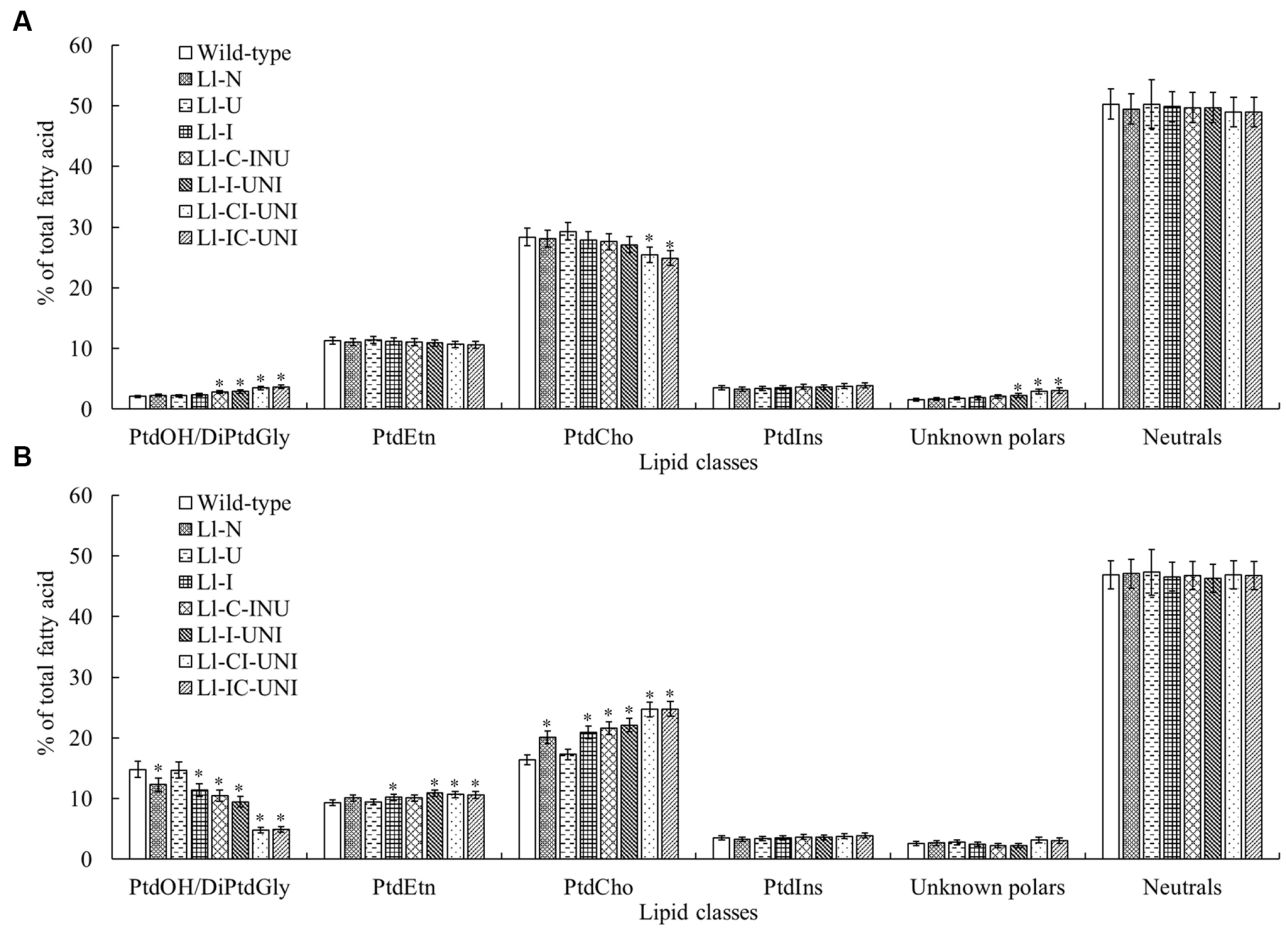
Alterations in proportions of main polar lipid classes for the 7 mutants and the wild-type isolate of *L. lecanii*. Lipids were extracted from mycelia pre-incubated for 4 d in PDB liquid medium **(A)** without and **(B)** with 550 μg/mL propamocarb. Data were means ± SD (*n* = 3). Significances between means for each mutant and the wild-type isolate were shown as ^*^ *p* < 0.05 according to the LSD test. Lipid abbreviations:DiPtdGly, diphosphatidylglycerol (cardiolipin); PtdCho, phosphatidylcholine; PtdEtn, phosphatidylethanolamine; PtdIns, phosphatidylinositol; PtdOH, phosphatidic acid.

**Figure 5:**
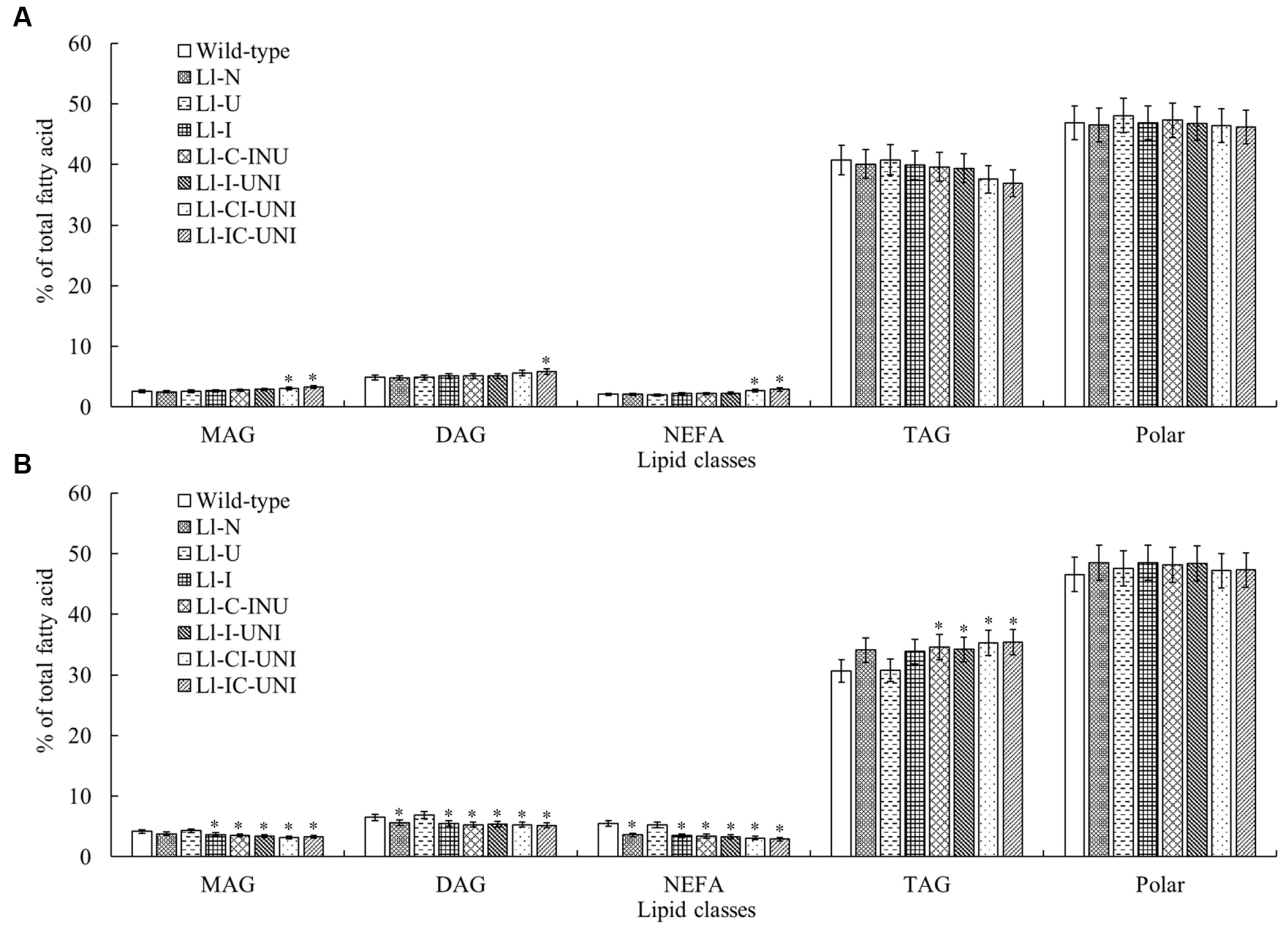
Alterations in proportions of main nonpolar lipid classes for the 7 mutants and the wild-type isolate of *L. lecanii*. Lipids were extracted from mycelia pre-incubated for 4 d in PDB liquid medium **(A)** without and **(B)** with 550 μg/mL propamocarb. Data were means ± SD (*n* = 3). Significances between means for each mutant and the wild-type isolate were shown as ^*^p < 0.05 according to the LSD test. Lipid abbreviations:DAG, diacylglycerol; MAG, monoacylglycerol; NEFA, nonesterified fatty acid; TAG, triacylglycerol.

The above 7 mutants (Ll-N, Ll-U, Ll-I, Ll-C-INU, Ll-I-UNI, Ll-CI-UNI, and Ll-IC-UNI) and the wild-type isolate were examined extracts for individual polar and neutral lipid classes. Major polar lipid and neutral lipid was phosphatidylcholine (PtdCho) (nearly 30%) and triacylglycerol (TAG) (nearly 40%), respectively. The proportion of total polars compared with total neutrals was significantly different between the mutants and the wild-type isolate (*F* = 3.71, df = 32, *p* < 0.01), and alternations in patterns of both groups were found in all samples untreated and treated by propamocarb (Figures 4, 5). Without propamocarb, the mutants excepting Ll-U had slightly lower PtdCho and higher PtdOH and unknown polars than the wild-type isolate for polar lipids (Figure 4A), and had slightly lower TAG and higher monoacylglycerol (MAG) and diacylglycerol (DAG) than the wild-type isolate for neutral lipids (Figure 5A). Under propamocarb stress, the most noticeable changes were an increase in PtdCho and a decrease in PtdOH for polar lipids (Figure 4B), and an increase in TAG and decreases in nonesterified fatty acid (NEFA), MAG and DAG for neutral lipids (Figure 5B). However, rangeability of lipid amounts was less in the mutants than that of the wild-type isolate to different extent. Rangeability of lipid amounts was the least and even tiny in the Ll-IC-UNI.

### Control against aphids

**Figure 6:**
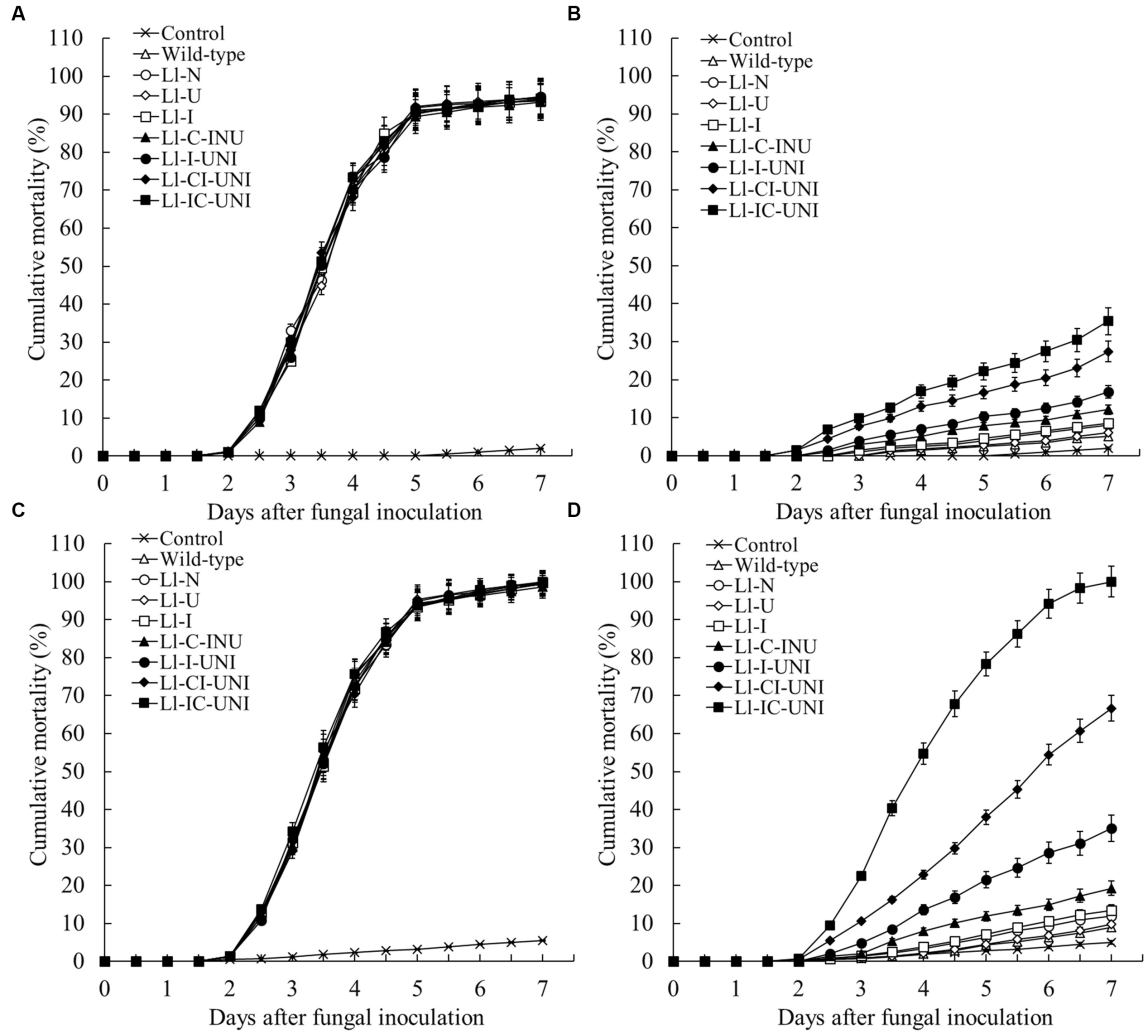
Cumulative mortality curves of adult cotton aphids treated with the 7 mutants and the wild-type isolate of *L*. *lecanii*. Conidial suspension (10^5^ conidia/ml) plus water control were applied on day 0 after spraying **(A)** water or **(B)** propamocarb and on day 3 after spraying **(C)** water or **(D)** propamocarb. Propamocarb was sprayed at the filed recommended dose of 550 μg/mL. Data were means ± SD (*n* = 3).

Without application of propamocarb, there was not significant difference in cumulative mortality of aphids by applying the conidial suspension (10^5^ conidia/mL) of each mutant and the wild-type isolate after spraying water (0-th day:*F* = 1.51, df = 240, *p* = 0.223; 3-th day:*F* = 1.42, df = 240, *p* = 0.462) (Figures 6A,C). Under propamocarb stress, interval time of applying *L*. *lecanii* after spraying propamocarb affected control efficacy against aphids (Figures 6B,D). When conidial suspension (10^5^ conidia/ml) were used immediately after spraying propamocarb, cumulative mortalities of aphids were always low (< 6%) until the end of 7-day observation in both the Ll-U and the wild-type isolate which were close to water control, and ranged from 8.0 to 16.8% in the other 4 mutants (Ll-N, Ll-I, Ll-C-INU and Ll-I-UNI) which were slightly higher than the wild-type isolate (Figure 6B). Only the Ll-CI-UNI and Ll-IC-UNI had more than 25.0% cumulative mortality of aphids, and they began to be significantly higher than those of other mutants from the 3th day (*F*_7,16_ = 476.00, *p* < 0.001). When conidial suspension were used 3 days after spraying propamocarb, cumulative mortalities of aphids still keep a low level (< 10%) till the 7th day for both the Ll-U and the wild-type isolate, and ranged from 12.0 to 19.2% for the 3 mutants (Ll-N, Ll-I, and Ll-C-INU) (Figure 6D). The other 3 mutants (Ll-I-UNI, Ll-CI-UNI and Ll-IC-UNI) had more than 35.0% cumulative mortality of aphids which was 35.1, 66.7, and 100.0%, respectively, at the end of 7-day observation, and they became significantly higher than other mutants from the 3th day (*F* = 942.60, df = 16, *p* < 0.001). The Ll-IC-UNI had an almost same curve of the cumulative mortality of aphids as the curve shown by the isolates without propamocarb.

## Discussion

Random single-agent mutagenesis of the “N,” “U,” and “I” in entomopathogenic *L. lecanii* enhanced propamocarb-tolerance 2.08, 1.13 and 2.43 times to the wild-type isolate, respectively. TR results for the“N” and “I” in *L. lecanii* were near to 2.05 and 2.39 in nematophagous *L. attenuatum* (Xie et al., 2017, 2018b). In previous studies, the highest mutation frequency obtained by NTG was associated with survival rates ranging from 0.6 to 16% in *Coprinus lagopus* (Moore, 1965), and ^2^C^5+^ ion-beam irradiation with survival rates ranging from 1 to 10% in *Saccharomyces cerevisiae* (Matuo et al., 2006), *Aspergillus oryzae* (Toyoshima et al., 2012) and *I. fumosorosea* (Shinohara et al., 2013). I and N gave the highest positive mutation rate at the survival rate of 12.8 and 21.2%, respectively, (Table 1). Certain differences in the relationship of positive mutation rates and survival rates were possibly attributed to response difference of *L. lecanii* cells. The previous fungal propamocarb tolerance was still not satisfying, but significant accumulation in propamocarb-tolerance of the mutants were found in multiple-round treatment of both the “N” and “U” while not in multiple-round treatment of the “I” (Xie et al., 2017, 2018a,b). As reported previously, the accumulative increase of fungal EC_50_ values stopped on the third-round “N” and the sixth-round “U” when the TRs were up to 2.12 and 2.79, respectively, (Xie et al., 2017, 2018b). These results indicate the difficulty of increasing fungal propamocarb tolerance which should not be conferred by single-point mutations of a gene, and confirm that propamocarb as a single-site inhibitor targeting in fatty acids has low to medium risk (FRAC Code List ©*, 2021). It was speculated that accumulative effects may be attributed to more sites mutated in *L. attenuatum* with the increase of treatment rounds both in the “N” and “I” (Xie et al., 2017, 2018b). Existing knowledge implied some potential mechanisms of mutagenic agents, for instance that UV light induced formation of pyrimidine dimers (Ikehata and Ono, 2011; Bose, 2014), NTG induced point mutagenesis from “G–C” to “A–T” (Li et al., 2013), and N^+^ iron-beam exerted the direct action of etching and damage of momentum transferring and the indirect action of the free radical of energy deposition (Song and Yu, 2000). In this study, we proposed a hypothesis that combined use of several mutagenic agents permutated properly is more efficient for improving the fungicide-tolerance of biocontrol fungi.

Mutagenesis modes and mutagenic agent permutations seem simple but are significant. Through gradual factorization of the increment of EC_50_ value of each mutant and analysis of the contributive rate of each agent, we found that the highest efficient agent was I/N/U significantly higher than I, N, and U (Table 2). I/N/U performed with a consecutive of I, N, and U, was not a simple single agent but a “novel” agent which maybe take advantages of complementation among different agents, and consequently was the best agent. Excellent mutants should be obtained more easily in mutagenesis with higher positive mutation rate. In this study, efficiency of enhancing propamocarb tolerance of mutants was the highest when the “U + N + I + I/N/U” was applied. However, this best mutagenesis mode only gave a medium high positive mutation rate of 10.4% (Table 1). Thus positive mutation rate, just one of many indicators, should be used cautiously in assessing mutagenic efficiency. The Ll-IC-UNI had the highest EC_50_ value of 3576.4 μg/mL and its TR reached to 7.1 in the mutagenesis mode of “U + N + I + I/N/U”(Figure 1). This propamocarb-tolerance level was 3 times more than the highest level in the “U” previously (Xie et al., 2018a). Overall, mutagenic effects on fungal propamocarb tolerance became more significant with increasing agent number, and were better in the intermittent mode although enhancement of propamocarb tolerance was relatively limited in two-agent mutagenesis. Interestingly synergistic effects were found in the “N + U,” “U + N,” “U + I,” U + N + I” and “U + N + I + I/N/U” (Table 2). Although the “N + U” and “U + N” had higher CTC, the “U + N + I + I/N/U still was the best mutagenesis mode for considering of synergistic effects and generation of the highest propamocarb-tolerant mutant. These results verified our hypothesis that combined use of several mutagenic agents permutated properly is more efficient in improving fungicide tolerance of a biocontrol fungus. Different from necessary of composite mutagenesis in *L. lecanii*, for another entomopathogenic fungus *I. fumosorosea*, combined irradiation may be unnecessary as the increase in benzimidazole fungicide resistance obtained separately with either ^2^C^5+^ and ^60^Co ion-beam irradiation method alone was considerable (Shinohara et al., 2013). A possible reason was that benzimidazole high-resistance of isolates and mutants were conferred by the replacement of amino acids at codon 198 and/or 200 in the β-tubulin locus (Schmidt et al., 2006) or by the mutations in the promoter region of the ABC transporter gene (ifT1) (Song et al., 2011b). We have to admitted that it is complicated and unclear why the accumulative effects in previous studies and the synergistic effects in the present study because of lacking information about mutation sites and their specific contribution on the increment of propamocarb tolerance.

Applicable mutants require not only propamocarb tolerance, but also stable heredity with normal biological characteristic. Propamocarb tolerance as indicated by EC_50_ value of 9 out of 23 mutants produced by single- and composite-mutagenesis including N^+^ ion-beam showed a significant increase during the first five generations, while finally recovered initial level of each mutant (Table 3). It was speculated in a previous report that limited damage to fungi by low-energy and low-dose ion-beam irradiation had been recovered during the period of continuous passaging, and then physiological recovery of the mutants occurred (Xie et al., 2018b). This physiological recovery might be accounted for increase of propamocarb tolerance of the mutants during the first five generations in this study. For biological characteristics, only 4 out of 31 mutants showed significant changes in colony growth on plates, sporulation on plates and aphids, and LC_50_ to aphids in laboratory (Table 4). Compared to the U-60-C6M in a previous study (Xie et al., 2018b), the Ll-IC-UNI had a faster mycelial growth rate, stronger sporulation capacity and higher virulence to aphids.

Action mode of propamocarb was to damage cell membrane permeability through affecting fatty acids biosynthesis and to finally inhibited mycelial growth, sporulation and germination of Oomycota (FRAC Code List ©*, 2021). Thus, it would be more convincing that lipid pattern/amount and cell membrane permeability were introduced into mutagenic-effect assessment combining analysis in target phenotypic traits to be improved and key biological characteristics. As reported previously, main constituents of cell membrane in *Phytophthora infestans* were obviously affected by a mandelamide pesticide (Griffiths et al., 2003), both polar and neutral lipids were noticeably changed in *L. lecanii* with the presence of propamocarb in this study (Figure 4B and Figure 5B), such as decrease of PtdCho and increase of PtdOH for polar lipids and decrease of TAG and increases of NEFA, MAG and DAG for neutral lipids. These alternations were less in the mutants and became slight with propamocarb-tolerance rise. Correspondingly damage of cell membrane permeability and final inhibition in fungal biomass in liquid medium with the presence of propamocarb were also reduced (Figures 2B, 3B).

Control of aphids by the mutants after applying propamocarb was also determined to explore potential use of the improved mutants. The interval time of applying *L. lecanii* was significantly shorten in the mutants after spraying propamocarb at 550 μg/mL. The extent of shorting time was greatly increasing with propamocarb-tolerance rise (Figure 6D). The Ll-IC-UNI, when applied on day 0 after spraying 550 mg/mL propamocarb, provided more than 27.5% cumulative mortality of aphids from the 6th day, while the U-60-C6M when applied on day 3 after spraying 550 mg/mL propamocarb only had 10.0% cumulative mortality of aphids till the 6th day (Xie et al., 2018b). Improved biological control agents might be almost used simultaneously with applying fungicides to control pest insects and crop diseases. Dosage of chemical insecticides would be reduced and pollution of these agrochemicals to agricultural environment would be relieved.

## Conclusion

Enhancement of propamocarb-tolerance in entomopathogenic *L. lecanii* is crucial to implement the coordinated application of fungal products and propamocarb formulations in order to achieve simultaneous prevention of both diseases and pests. In this study, we constructed various composite mutagenesis modes based on UV-light, NTG and low energy N^+^ ion-beam, and then investigated their performance of improving propamocarb tolerance and maintaining excellent biological characteristic in *L. lecanii*. Overall, the ‘U + N + I + I/N/U″ was highest efficient to improve the propamocarb-tolerance of *L. lecanii* and the obtained Ll-IC-UNI could have commercial potential for field application. Our findings indicate that mutants with different propamocarb-tolerance may be generated by various mutation loci from different action mode of mutagenic agents, and such mutants will be useful for the study of underlying mechanism of propamocarb-resistance in future.

## Data availability statement

The original contributions presented in the study are included in the article/supplementary material, further inquiries can be directed to the corresponding author.

## Author contributions

YZ conceived and designed the study. YZ, XZ, and WQ performed the experiments. YZ and XZ interpreted the data and wrote the manuscript. All authors read and approved the final version of the manuscript.

## Funding

This research was supported by the National Natural Science Foundation of China (Grant 32271651) and the Chinese academy of Agricultural Sciences Innovation Program (Agro-Environmental Protection Institute).

## Conflict of interest

The authors declare that the research was conducted in the absence of any commercial or financial relationships that could be construed as a potential conflict of interest.

## Publisher’s note

All claims expressed in this article are solely those of the authors and do not necessarily represent those of their affiliated organizations, or those of the publisher, the editors and the reviewers. Any product that may be evaluated in this article, or claim that may be made by its manufacturer, is not guaranteed or endorsed by the publisher.
